# Unraveling the Complexity: Ehrlichiosis-Induced Septic Shock and Acute Respiratory Distress Syndrome

**DOI:** 10.7759/cureus.57682

**Published:** 2024-04-05

**Authors:** Anshu Kataria, Anne M Arcidiacono, Minhaz Murshad, Shuvendu Sen, Ahmad A Homoud

**Affiliations:** 1 Internal Medicine, Jersey Shore University Medical Center, Neptune, USA; 2 Pulmonary and Critical Care Medicine, Jersey Shore University Medical Center, Neptune, USA

**Keywords:** ehrlichia chaffeensis, septic shock [ss], tick-borne diseases, severe sepsis, acute respiratory distress syndrome [ards], ehrlichiosis

## Abstract

Human monocytic ehrlichiosis typically presents with nonspecific cold-like symptoms and a history of recent tick exposure, often responding well to early treatment. Here, we present the case of a 67-year-old immunocompetent male who initially presented with fevers, chills, dysuria, and hematuria, leading to admission to the intensive care unit with septic shock and acute respiratory distress syndrome (ARDS), which was later attributed to *Ehrlichia chaffeensis* infection. Prompt treatment with doxycycline resulted in a full clinical recovery. This case highlights the rare occurrence of severe ehrlichiosis and provides insights into its effective management based on updated literature.

## Introduction

Ehrlichiosis is a disease transmitted by tick-borne vectors, specifically the lone star and blacklegged ticks found primarily in the northeast and southeast regions of the USA [1, 2]. Cases of ehrlichiosis have been steadily rising since the year 2000 in the USA, with concern that it may be linked to warmer climate change [3]. Onset typically occurs five to 14 days after the initial tick bite, with early illness presenting with mild nonspecific symptoms, i.e., fevers/chills, headache, malaise, myalgias, and rash (mostly in children). According to the Centers for Disease Control and Prevention (CDC), late illness is typified by severe manifestations of CNS involvement (20%), acute respiratory distress syndrome (ARDS), septic shock, toxic shock-like syndromes, renal/hepatic failure, and coagulopathies. The CDC also states that risk factors for these severe manifestations include delayed treatment, age extremes, and immunocompromising conditions [4]. The treatment for ehrlichiosis is typically doxycycline for seven to 10 days with favorable outcomes and a rare progression into late/severe illness.

## Case presentation

Investigation

We report the case of a 67-year-old male patient with a medical history of hyperlipidemia, nonobstructive coronary artery disease, and benign prostatic hyperplasia who presented with a high-grade fever, hematuria, dysuria, lethargy, and increased urinary frequency, initially suspected to be prostatitis-related sepsis. The patient had recently traveled to Niagara Falls and Toronto and had been prescribed nitrofurantoin without symptom improvement. Notably, the only other medication the patient was prescribed was tamsulosin for benign prostatic hyperplasia, which he had not started taking.

Upon arrival at the ED, the patient's temperature was 100.6°F, and he displayed tachycardia at 104 beats per minute with a blood pressure of 119/78 mmHg. During his ED stay, his temperature spiked to 104.3°F, and he became hypotensive at 86/48 mmHg. Physical examination revealed rigors, sinus tachycardia, and diaphoresis.

Diagnosis

Initial laboratory findings included thrombocytopenia (platelet count of 37), an elevated segmented neutrophil percentage (76.3%), and transaminitis, with elevated levels of aspartate aminotransferase (153 U/L), alanine transaminase (174 U/L), and alkaline phosphatase (179 U/L), as shown in Table 1. Urinalysis showed numerous RBCs and proteins without WBCs. CT abdomen and pelvis showed an enlarged prostate. Given the transaminitis, a right upper quadrant ultrasound and hepatobiliary iminodiacetic acid scan were performed, both of which ruled out cholecystitis.

**Table 1:** Relevant lab findings. H: high; L: low.

Lab values	Initial labs	At the time of the development of septic shock	Reference values
Prothrombin time	-	14.7 (H)	11-13.5 seconds
International normalized ratio	-	1.26 (H)	0.9-1.1
Partial thromboplastin time	-	46 (H)	25-35 seconds
Fibrinogen	-	505	200-400
WBCs	4.6	10.8	4.5-11.0 x 10^3^/uL
Hemoglobin	13.3	11.1 (L)	13.5-17.5 g/dL
Hematocrit	39.6	33.0 (L)	41.0-53.0%
Platelet count	37	25 (L)	150-450 x 10^3^/uL
Lymphocytes, %	4.4 (L)	2.6 (L)	20-40%
Monocytes, %	5.3	1.8	2-10%
Eosinophils, %	0.0	0.0	1-6%
Neutrophils, absolute	4.0	9.0 (H)	2-7 x 10^3^/uL
Lymphocytes, absolute	0.3 (L)	0.4 (L)	1-4 x 10^3^/uL
Monocytes, absolute	0.2	0.2	0.2-0.8 x 10^3^/uL
Eosinophils, absolute	0.0	0.0	0.04-0.4 x 10^3^/uL
Segmented neutrophils, %	76.3 (H)	26.3 (L)	40-75%
Bands, %	10.5	57.0 (H)	0-5%
Metamyelocytes	0.9 (H)	11.4 (H)	0.0%
Metamyelocytes, absolute	0.0	1.2 (H)	0.0 x 10^3^/uL
Anisocytosis	1+ (Slight)	1+ (Slight)	Normal
Macrocytes	1+ (Slight)	1+ (Slight)	Normal
RBC morphology	Abnormal	Abnormal	Normal
Glucose	149 (H)	65 (L)	70-100 mg/dL
Blood urea nitrogen	12	12	7-20 mg/dL
Creatinine	1.14	1.07	0.6-1.2 mg/dL
Estimated glomerular filtration rate	≥60	≥60	>60 mL/min/1.73 m^2^
Sodium	133 (L)	140	135-145 mmol/L
Chloride	101	108 (H)	98-107 mmol/L
Anion gap	7	9	5-15 mmol/L
Calcium	8.8	7.7 (L)	8.7-10.4 mmol/L
Bicarbonate	25	23	20-31 mmol/L
Alkaline phosphatase	179 (H)	133 (H)	46-116 U/L
Protein total	6.8	4.9 (L)	6.0-8.3 g/dL
Albumin	3.3 (L)	2.0 (L)	3.4-5.0 g/dL
Bilirubin total	1.0	1.3	0.2-1.3 mg/dL
Aspartate aminotransferase	153 (H)	345 (H)	0-34 U/L
Alanine transaminase	174 (H)	258 (H)	10-49 U/L
Brain natriuretic peptide	-	155 (H)	<100 pg/mL
Lactic acid	1.7	2.7 (H)	0.5-2.2 mmol/L
Procalcitonin	1.02 (H)	7.32 (H)	<0.50 ng/mL
Lactate dehydrogenase	-	516 (H)	120-246 U/L
Ferritin	-	1502.2 (H)	10.5-307.3 ng/mL
Haptoglobin	-	106	40-280 mg/dL

**Figure 1:**
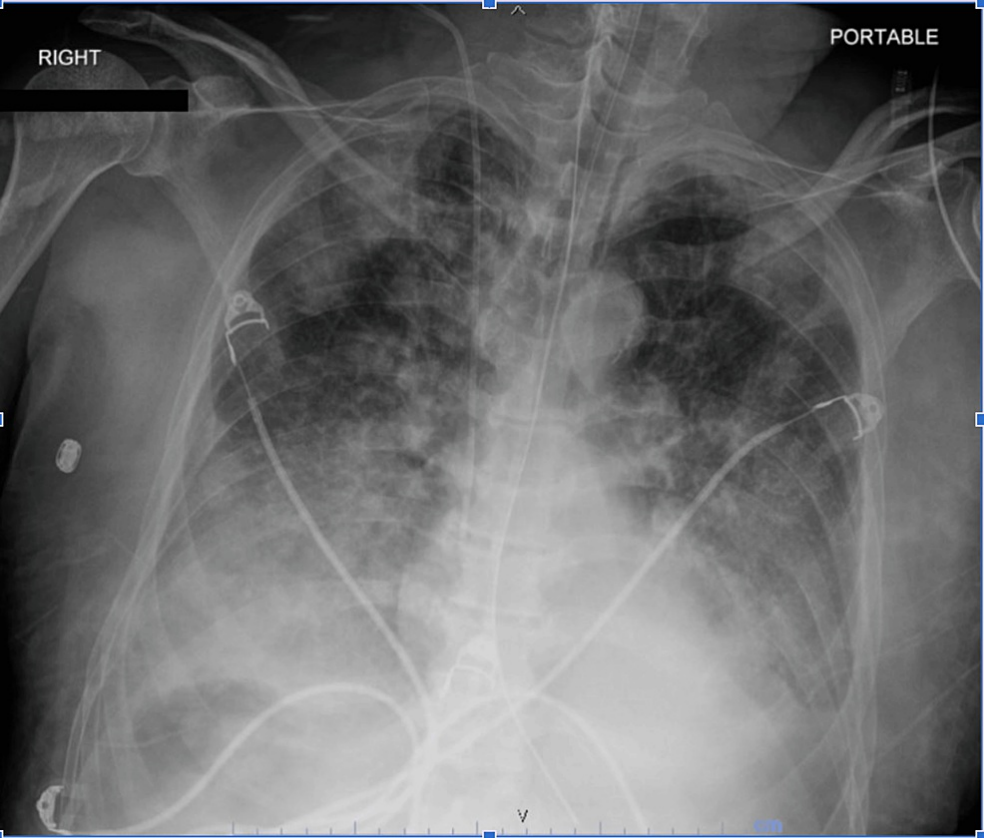
Chest X-ray demonstrating ARDS in our patient. ARDS: acute respiratory distress syndrome.

With the patient's clinical presentation and laboratory findings, sepsis treatment was initiated, focusing on the prostate as the source (suspected prostatitis). IV vancomycin and IV piperacillin/tazobactam were administered, and the patient was transferred to the intensive care unit (ICU) due to concern for septic shock. During the ICU stay, the patient's condition deteriorated, with increasing pressor requirements, rising lactic acidosis, intermittent temperature spikes, and the development of ARDS requiring intubation. Infectious disease specialists recommended broadening antibiotic coverage for better prostate penetration, resulting in the addition of IV levofloxacin. On the fourth day of admission, a positive *Ehrlichia chaffeensis* PCR confirmed the diagnosis (Figure 1).

Treatment

The patient received prompt IV doxycycline (100 mg twice daily for one week) and prior antibiotics were discontinued, leading to decreased pressor requirements and an improved overall condition.

Follow-up and outcomes

Eleven days after recovering from ARDS, the patient was successfully extubated. During his hospitalization, he experienced speech and swallowing difficulties necessitating G-tube placement. Otherwise, his hospital course was unremarkable, and he was discharged to subacute rehabilitation with a full recovery.

## Discussion

Human monocytic ehrlichiosis (HME), as seen in our patient, is caused by *E. chaffeensis*, a Gram-negative intracellular bacterium transmitted by blacklegged and lone star ticks [5]. This bacterium has been increasingly detected in the northeastern and southeastern USA. *Ehrlichia chaffeensis* invades phagocytes through endocytosis, suppressing apoptosis and inhibiting innate immune responses, primarily affecting peripheral blood cells [5]. Though not tried in our case, interferon (IFN)-gamma is key to clearing *E. chaffeensis* infection, and iron chelation therapy with deferoxamine can inhibit its propagation [6].

Ehrlichiosis symptoms vary widely, with most clinical manifestations resulting from the host's inflammatory response rather than the bacterium itself. It is often likened to a "toxic shock-like syndrome" due to the robust immune response despite low bacterial loads. Common initial symptoms include fever, chills, severe headache, myalgias, nausea, vomiting, diarrhea, loss of appetite, and confusion, with laboratory findings such as leukopenia, thrombocytopenia, and transaminitis. Approximately one-third of patients may develop a rash, typically appearing around five days after fever onset [7].

Early detection and treatment are critical for preventing the progression to severe disease. Given the nonspecific symptoms and frequent lack of tick bite recollection [8], diagnosis often relies on PCR, as *Ehrlichia* morulae are rarely seen in peripheral smears. Doxycycline is the primary treatment, with few reports of success with rifampin and only in in vitro studies [9,10]. Patients typically respond to doxycycline within 48-72 hours.

Prevention is essential, focusing on avoiding tick bites. Post-tick bite prophylaxis and testing patient-retrieved ticks for infection are not recommended [11]. The CDC outlines guidelines for tick-borne disease prevention, including avoiding tick-infested areas, wearing protective clothing, applying permethrin spray, using Environmental Protection Agency-registered insect repellents, and conducting thorough skin checks after outdoor activities [12].

## Conclusions

The number of ehrlichiosis cases has been steadily rising, necessitating consideration of this diagnosis in endemic areas. While the case fatality rate remains low, the risk of progression to severe disease underscores the importance of early recognition and treatment. Our case emphasizes the need for a broad differential diagnosis when dealing with a fever of unknown origin or when conventional sepsis treatment is ineffective. Further research is warranted to develop more rapid testing and detection methods.

## References

[1] (2023). Approximate distribution of the lone star tick. https://www.cdc.gov/ticks/maps/lone_star_tick.html.

[2] (2023). General distribution of blacklegged ticks (Ixodes scapularis). https://www.cdc.gov/ticks/maps/blacklegged_tick.pdf.

[3] Bouchard C, Dibernardo A, Koffi J, Wood H, Leighton PA, Lindsay LR (2019). Increased risk of tick-borne diseases with climate and environmental changes. Can Commun Dis Rep.

[4] (2023). Centers for Disease Control and Prevention. Ehrlichiosis. http://www.cdc.gov/Ehrlichiosis/.

[5] McBride JW, Walker DH (2023). Molecular and cellular pathobiology of Ehrlichia infection: targets for new therapeutics and immunomodulation strategies. Expert Rev Mol Med.

[6] Barnewall RE, Rikihisa Y (2023). Abrogation of gamma interferon-induced inhibition of Ehrlichia chaffeensis infection in human monocytes with iron-transferrin. Infect Immun.

[7] (2023). Ehrlichiosis: signs and symptoms. https://www.cdc.gov/ehrlichiosis/symptoms/index.html.

[8] (2023). RMSF: deadly, but preventable. https://www.cdc.gov/ncezid/dvbd/media/rmsf.html.

[9] Branger S, Rolain JM, Raoult D (2004). Evaluation of antibiotic susceptibilities of Ehrlichia canis, Ehrlichia chaffeensis, and Anaplasma phagocytophilum by real-time PCR. Antimicrob Agents Chemother.

[10] Abusaada K, Ajmal S, Hughes L (2016). Successful treatment of human monocytic ehrlichiosis with rifampin. Cureus.

[11] (2023). Tickborne diseases: diagnosis and management. https://www.aafp.org/pubs/afp/issues/2020/0501/p530.html.

[12] (2023). Preventing tick bites on people. https://www.cdc.gov/lyme/prev/on_people.html.

